# Gold Inlays; an Easy Method

**Published:** 1906-04

**Authors:** W. A. Capon


### GOLD INLAYS; AN EASY METHOD.

By W. A. Capon, D. D. S.

(Clinic before Pennsylvania State Dental Society, Philadelphia, June, 1905.)

This clinic was given to demonstrate that such operations are not only practical, but desirable, with the advantage of being easily and quickly performed and is described as follows: The cavities are prepared in the manner usual for porcelain inlays and a matrix is made either of gold or platinum foil, same as used for porcelain, although heavier can be used with no disadvantage. While the matrix is in the cavity mat gold is packed into it in much the same manner as spunk. No particular care is necessary and dampness is not harmful. The gold may be of any manufacture provided it is of the mat form. It is used as a medium for soldering and also answers the purpose of covering any break that may occur in the matrix.

When the cavity is partly filled with gold, remove the matrix and cover the exposed surface with liquid rouge, whiting or fine ochre, carefully tracing with small pencil brush to edge of cavity, thereby preventing solder from flowing anywhere except in cavity. The form is now laid on soldering block and the packed mat gold touched with a little liquid soldering flux and small pieces of 22K. solder melted into the gold until cavity is full. If to be contoured small pieces or melted pellets of gold plate are placed at proper points. The excess matrix material is now trimmed away and the filling tried in cavity and occlusion noted.

It is then partially finished and cemented in place, using force if necessary. After the cement is hard the filling is polished in the usual manner.

It is not necessary that any matrix should be invested and when gold is used it is protected from fusing by coating every portion of the matrix except the cavity proper, thus resisting heat to an astonishing degree.

It is recommended that rouge is the preferable substance to protect the matrix because of its great fineness and affinity for a smooth surface. This material is bought in powder and spatulated with alcohol and water.—*Items of Interest.*

---

